# What is the impact of AI-driven prosthodontic planning on dental implant positioning outcomes? A systematic review

**DOI:** 10.1007/s44445-026-00184-6

**Published:** 2026-06-01

**Authors:** Dugagjin Sokoli, Burim Kiseri, Genc Demjaha, Flora Asllani-Hoxha, Venera Bimbashi

**Affiliations:** 1 University of Business and Technology, Prishtina, Kosovo; 2 University of Prishtina “Hasan Prishtina”, Prishtina, Kosovo; 3 Department of Prosthodontics, University Dentistry Clinical Center of Kosovo, Prishtinë, Republic of Kosovo; 4 Department of Prosthodontics Alma Mater Europaea, Campus College Rezonanca, Prishtinë, Republic of Kosovo

**Keywords:** Artificial intelligence, Prosthodontics, Dental implants, Implant positioning, Machine learning, Predictive analytics

## Abstract

Purpose: This systematic review aimed to evaluate the role of artificial intelligence (AI)–based technologies, including machine learning algorithms, predictive analytics, and AI-assisted surgical guidance, in prosthodontic-driven planning for dental implant positioning, with particular attention to diagnostic accuracy, planning consistency, and workflow optimization. Methods: This systematic review aimed to evaluate the role of artificial intelligence (AI)-based technologies, including machine learning algorithms, predictive analytics, and AI-assisted surgical guidance, in prosthodontic-driven planning for dental implant positioning, with particular attention to diagnostic accuracy, planning consistency, and workflow optimization. Results: The included studies suggest that AI-based approaches are associated with improved consistency in diagnostic image interpretation, enhanced accuracy of virtual implant planning, and reduced operator-dependent variability. Across predominantly moderate- to high-quality studies, AI-assisted workflows were reported to support more individualized implant positioning by integrating anatomical, prosthetic, and occlusal parameters. However, the evidence remains heterogeneous, and reported benefits are primarily derived from observational and pilot studies rather than large-scale clinical trials. Conclusions: Current evidence indicates that AI has the potential to support prosthodontic-driven implant planning by enhancing diagnostic standardization and facilitating personalized treatment strategies. Nevertheless, the absence of quantitative synthesis, limited external validation of AI models, and variability in study quality warrant cautious interpretation. Further well-designed clinical studies and standardized validation protocols are required before definitive conclusions regarding clinical outcomes can be drawn.

## Introduction

The integration of artificial intelligence (AI) into prosthodontic planning has emerged as a notable development in contemporary dental implantology, with increasing attention to precision, efficiency, and treatment predictability (Lee et al. 2019; Yang et al. 2020). Within prosthodontics, accurate implant positioning is essential for achieving long-term functional stability and esthetic harmony. Dental implants, as a fundamental component of prosthodontic rehabilitation, require precise planning to ensure biomechanical compatibility and prosthetic success (Zhang and Li 2018). Conventionally, implant positioning has depended on clinician expertise and manual interpretation of diagnostic images. Despite advances in digital imaging and planning technologies, such approaches remain susceptible to operator-dependent variability, which may contribute to complications such as prosthetic misalignment, nerve injury, peri-implantitis, or implant failure (Patel et al. 2020).

AI-driven prosthodontic planning represents a shift from exclusively experience-based decision-making toward data-supported clinical workflows through the application of machine learning algorithms, neural networks, and predictive analytics (Kim et al. 2017; Yasui et al. 2021). These technologies enable the processing of complex diagnostic datasets, including three-dimensional imaging, cone-beam computed tomography (CBCT), intraoral scans, and patient-specific anatomical information (Lee et al. 2019; Yang et al. 2020; Yasui et al. 2021). By integrating AI into prosthodontic workflows, treatment planning may become more standardized, with reduced inter-operator variability and improved reproducibility of planning outcomes (Roberts and Adams 2016). Importantly, AI-assisted systems are intended to complement rather than replace clinician judgment, functioning primarily as decision-support tools (Yasui et al. 2021; Chen et al. 2019).

A central advantage of AI-assisted prosthodontic planning lies in its potential to enhance diagnostic consistency and reduce subjective interpretation (Yang et al. 2020; Wang et al. 2020). AI-based image recognition and segmentation systems, particularly those employing deep learning architectures, facilitate detailed analysis of CBCT data and radiographic images. These tools support the identification of anatomical landmarks, assessment of bone quality, and evaluation of spatial constraints relevant to implant positioning (Lee et al. 2019; Liu et al. 2020). Through preoperative simulation of multiple implant placement scenarios, AI-based planning systems allow clinicians to compare alternatives and select implant positions that best align with prosthetic and functional objectives (Kim et al. 2017; Yasui et al. 2021).

Beyond technical accuracy, AI contributes to the evolving concept of precision dentistry, in which treatment strategies are increasingly tailored to individual patient characteristics (Zhang and Li 2018; Johnson and Blake 2018). Traditional implant protocols often rely on standardized guidelines that may not adequately reflect patient-specific anatomical and biomechanical variability. In contrast, AI-based planning systems can integrate parameters such as bone morphology, soft tissue characteristics, occlusal force distribution, and biomechanical considerations to support individualized implant positioning (Yang et al. 2020; Kim et al. 2017; Johnson and Blake 2018). This personalized approach may help reduce occlusal discrepancies, prosthetic misalignment, and excessive loading forces that could compromise prosthetic performance or implant longevity (Zhang and Li 2018; Garcia et al. 2020).

Beyond technical accuracy, AI contributes to the evolving concept of precision dentistry, in which treatment strategies are increasingly tailored to individual patient characteristics (Zhang and Li 2018; Johnson and Blake 2018). Traditional implant protocols often rely on standardized guidelines that may not adequately reflect patient-specific anatomical and biomechanical variability. In contrast, AI-based planning systems can integrate parameters such as bone morphology, soft tissue characteristics, occlusal force distribution, and biomechanical considerations to support individualized implant positioning (Yang et al. 2020; Kim et al. 2017; Johnson and Blake 2018). This personalized approach may help reduce occlusal discrepancies, prosthetic misalignment, and excessive loading forces that could compromise prosthetic performance or implant longevity (Zhang and Li 2018; Garcia et al. 2020).

This systematic review provides a comprehensive evaluation of AI-driven prosthodontic planning and its implications for dental implant positioning. Specifically, the review examines how AI technologies are being applied to support diagnostic precision, enhance virtual treatment planning, improve surgical execution, and facilitate prosthetic integration. By synthesizing findings from recent literature, the review aims to clarify the current role of AI within prosthodontic implantology and to assess its relevance to contemporary clinical workflows (Zhang and Li 2018; Reddy et al. 2019; Thomas et al. 2019).

In addition to technical and clinical considerations, AI-assisted planning has been associated with improvements in overall treatment workflow and patient experience. By supporting more predictable planning and reducing the need for corrective interventions, AI-based systems may contribute to greater efficiency in implant therapy (Garcia et al. 2020; Thomas et al. 2019). Furthermore, the use of digital simulations and visual planning tools may enhance patient communication and informed consent, potentially influencing treatment acceptance and patient confidence in implant procedures (Kim et al. 2017; Garcia et al. 2020).

Despite these potential advantages, the clinical integration of AI into prosthodontic practice is accompanied by several challenges. Ethical and practical concerns related to data privacy, algorithm transparency, and clinician reliance on AI-generated recommendations require careful consideration (Roberts and Adams 2016; Chen et al. 2019; Reddy et al. 2019). Moreover, the absence of standardized protocols for AI-assisted implant planning limits comparability across studies and complicates widespread clinical adoption (Roberts and Adams 2016; Zhou et al. 2020). Addressing these issues is essential to ensure safe and responsible implementation of AI technologies in prosthodontic implantology.

Ongoing research continues to refine AI-driven applications in prosthodontics, with efforts focused on improving algorithm robustness, validation, and clinical usability (Thomas et al. 2019; Hwang et al. 2020). However, many published studies remain exploration, involve limited sample sizes, or lack external validation, which may restrict the generalizability of reported findings. Consequently, the interpretation of AI-related outcomes should remain cautious, particularly in the absence of quantitative synthesis or long-term clinical trials (Simmons et al. 2017; Zhou et al. 2020).

Accordingly, the objective of this systematic review is to critically evaluate the current evidence on AI-assisted prosthodontic-driven implant planning, with emphasis on diagnostic consistency, planning accuracy, workflow integration, and methodological quality. By identifying both the potential contributions and the limitations of existing evidence, this review aims to inform clinicians and researchers about the current state of AI integration in prosthodontic implantology and to highlight priorities for future research (Kim et al. 2017; Yasui et al. 2021; Hwang et al. 2020; Singh and Patel 2019).

## Methods

### Protocol and registration

**Fig. 1:**
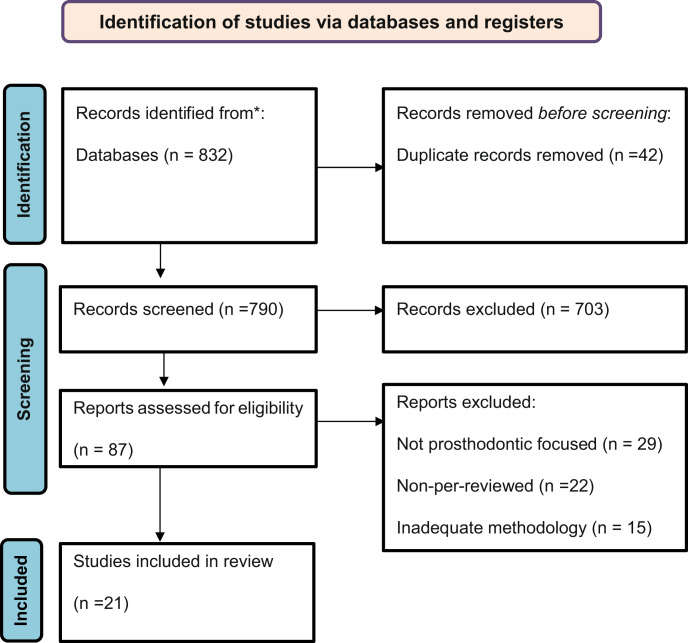
PRISMA flow diagram of studies discussing AI in prosthodontic implant planning

This systematic review was conducted to critically evaluate the role of artificial intelligence (AI) in prosthodontic-driven planning for dental implant positioning. The methodological approach was developed in accordance with established standards for evidence synthesis and followed the Preferred Reporting Items for Systematic Reviews and Meta-Analyses (PRISMA) 2020 guidelines to ensure transparency, reproducibility, and methodological rigor throughout all stages of the review process (Page et al. 2021). A PRISMA flow diagram was generated to illustrate the study selection process, including identification, screening, eligibility assessment, and final inclusion of studies (Figure 1).

The review protocol adhered to PRISMA 2020 recommendations, ensuring that eligibility criteria, data extraction procedures, quality appraisal, and qualitative synthesis were predefined and applied consistently. This systematic review was not registered in the PROSPERO database. The decision not to register the protocol was primarily related to the exploratory and interdisciplinary nature of the topic, which integrates prosthodontics, digital dentistry, and artificial intelligence. This omission is acknowledged as a methodological limitation with respect to protocol transparency. Consistent with PRISMA 2020 emphasis on transparent reporting, future studies should provide reproducible descriptions of AI pipelines and validation approaches to enable meaningful synthesis and implementation appraisal (Page et al. 2021).

### Database search and selection criteria

A comprehensive literature search was performed across four electronic databases, PubMed, Scopus, IEEE Xplore, and Google Scholar, covering publications from January 2010 to December 2024. These databases were selected due to their extensive coverage of medical, dental, engineering, and informatics literature, thereby enabling the identification of interdisciplinary studies relevant to AI applications in prosthodontics and implantology (Zhang and Li 2018; Garcia et al. 2020; Thomas et al. 2019).

The search strategy employed a structured combination of Medical Subject Headings (MeSH) and free-text keywords, including “Artificial Intelligence in Prosthodontics,” “AI in Dental Implantology,” “Machine Learning in Dentistry,” “AI-Assisted Implant Planning,” and “Prosthodontic Implant Positioning.” Boolean operators (AND/OR) were applied to enhance search sensitivity and specificity. Full database-specific search strings are provided in Appendix A to ensure reproducibility.

The initial search yielded a total of 832 records, which were exported to reference management software (EndNote) for duplicate removal. Following this process, the remaining records underwent a two-stage screening procedure. In the first stage, titles and abstracts were independently screened by two reviewers to exclude studies that did not meet the predefined research objectives. In the second stage, full-text articles were assessed for eligibility based on methodological quality, relevance to prosthodontic-driven implant planning, and compliance with the inclusion criteria. Any disagreements during the screening process were resolved through discussion and consensus; when consensus could not be reached, a third reviewer was consulted to reach a final decision, in accordance with PRISMA recommendations (Page et al. 2021).

### Inclusion criteria

To ensure scientific rigor and clinical relevance, predefined inclusion and exclusion criteria were applied to all identified studies. With respect to scope, only articles that specifically investigated the application of artificial intelligence (AI) in guiding implant positioning within a prosthodontic-driven context were considered eligible. Studies addressing AI in general dental applications without a direct link to prosthodontic-oriented implant planning were excluded to maintain clinical and conceptual focus (Zhang and Li 2018; Garcia et al. 2020; Hwang et al. 2020).

Studies examining AI applications explicitly within prosthodontic-driven implant planning were included. Only peer-reviewed journal articles published in English were considered eligible. Grey literature, conference abstracts without full manuscripts, and other non–peer-reviewed sources were excluded to ensure methodological consistency and reliability of the evidence base (Reddy et al. 2019; Thomas et al. 2019).

Regarding study design, the review included randomized controlled trials (RCTs), cohort studies, and case–control studies. Systematic reviews identified during the search process were not included in the qualitative synthesis of outcomes in order to avoid double-counting of primary data; instead, they were used solely to provide contextual background and to support interpretation of findings where appropriate. Qualitative studies were excluded unless they offered substantive insights into the clinical integration of AI technologies in prosthodontic implant planning (Thomas et al. 2019; Zhou et al. 2020).

Only studies published in English were included to ensure consistency in data interpretation. In addition, publication dates were restricted to the period between 2010 and 2024, capturing research aligned with the contemporary development and clinical application of AI technologies in dental medicine (Kim et al. 2017; Hwang et al. 2020).

Following the application of these criteria, 87 studies were shortlisted for full-text assessment. After detailed methodological appraisal, 21 studies met all inclusion requirements and were selected for final qualitative analysis.

### Data extraction and analysis

This systematic review was conducted in accordance with PRISMA 2020 guidelines to ensure transparency and reproducibility throughout the data extraction and synthesis process (Page et al. 2021). As previously noted, the review protocol was not registered in the PROSPERO database, which is acknowledged as a methodological limitation with respect to protocol transparency. Data extraction focused on predefined variables relevant to prosthodontic-driven implant planning. Initially, the primary objectives of each study were recorded, with attention to clearly stated aims and underlying hypotheses (Zhang and Li 2018; Garcia et al. 2020).

Subsequently, key methodological characteristics were extracted, including study design, type of AI model employed (e.g., machine learning algorithms, deep learning models, convolutional neural networks), sample size, clinical or experimental setting, and the specific prosthodontic intervention or planning phase addressed (Lee et al. 2019; Yasui et al. 2021; Liu et al. 2020).

Outcome data were then collected, with emphasis on reported measures such as implant positioning accuracy, planning consistency, workflow efficiency, and prosthetic-related outcomes. Where reported, observations related to complication rates or procedural deviations were documented; however, such findings were interpreted cautiously given the heterogeneity of study designs and the absence of quantitative synthesis (Patel et al. 2020; Kim et al. 2017; Simmons et al. 2017). Each study’s main conclusions were summarized, focusing on the authors’ interpretations and the clinical relevance of the findings within prosthodontic practice (Garcia et al. 2020; Thomas et al. 2019; Feng et al. 2021).

Finally, limitations reported by the original authors were extracted, including issues related to sample size, potential sources of bias, model validation, and generalizability of results (Simmons et al. 2017; Zhou et al. 2020). Data extraction and quality assessment were performed independently by two reviewers. Methodological quality was evaluated using the Newcastle–Ottawa Scale for observational studies and the Cochrane Risk of Bias tool for randomized studies. Any discrepancies were resolved through discussion and consensus, with consultation of a third reviewer when necessary.

## Results

A total of 21 studies met the inclusion criteria following full-text evaluation. The corrected PRISMA flow diagram (Figure 1) illustrates the study selection process. The included studies examined a range of artificial intelligence (AI) applications within prosthodontic-driven implant planning, encompassing diagnostic assessment, virtual treatment planning, surgical guidance, and prosthetic considerations. Overall, the synthesized evidence suggests that AI integration has been associated with improved consistency in diagnostic interpretation, enhanced accuracy of virtual implant positioning, and increased predictability of planning workflows. However, these findings should be interpreted in light of the heterogeneity of study designs and outcome measures.

This systematic review identified several key trends regarding the role of AI in prosthodontic-driven planning for dental implant positioning. Across the included literature, AI-based approaches were reported to support diagnostic standardization, assist in implant positioning accuracy, facilitate individualized treatment planning, and improve workflow organization. While some studies also reported reductions in intraoperative deviations or procedural inefficiencies, these observations were primarily derived from observational or pilot investigations rather than large-scale controlled trials. Table 1 provides a structured overview of AI applications across the major phases of prosthodontic implant treatment, summarizing reported functionalities, proposed clinical advantages, and supporting references.

### Synthesis of impact

The findings of this systematic review indicate that artificial intelligence (AI) may contribute to multiple aspects of prosthodontic-driven implantology, with potential implications for diagnostic accuracy, planning consistency, and procedural efficiency. Several studies reported that AI-assisted image analysis was associated with improved consistency in the identification of anatomical landmarks and pathological features when compared with conventional interpretation methods (Lee et al. 2019; Yang et al. 2020; Johnson and Blake 2018). These observations suggest a potential role for AI in reducing inter-operator variability during diagnostic assessment.

In addition, AI-driven planning systems were reported to support greater standardization and accuracy in virtual implant positioning, particularly by integrating anatomical and prosthetic parameters within digital workflows (Zhang and Li 2018; Yasui et al. 2021; Reddy et al. 2019). Predictive modeling techniques embedded in some AI platforms were described as enabling preoperative risk assessment and decision support, which may assist clinicians in anticipating procedural challenges (Kim et al. 2017; Simmons et al. 2017; Zhou et al. 2020). Furthermore, several studies highlighted the role of AI in facilitating personalized treatment planning through the incorporation of patient-specific anatomical, functional, and systemic data (Garcia et al. 2020; Hwang et al. 2020; Singh and Patel 2019).

Automation of diagnostic analysis, planning procedures, and, in some cases, manufacturing workflows was also reported to be associated with reductions in planning time and improved workflow efficiency (Patel et al. 2020; Reddy et al. 2019; Blake and Thompson 2018; Feng et al. 2021). However, quantitative estimates of time or cost savings were inconsistently reported and were often based on individual studies with limited sample sizes. Taken together, the available evidence suggests that AI may augment clinician decision-making and workflow organization in prosthodontic implantology, although the magnitude and clinical relevance of these effects remain dependent on study quality and context.

### Quality assessment

All 21 included studies underwent methodological quality assessment using validated appraisal tools. Because no randomized controlled trials met the eligibility criteria at full-text assessment, all included studies were appraised using the Newcastle–Ottawa Scale (NOS), focusing on selection of study cohorts, comparability between groups, and outcome assessment. The Cochrane Risk of Bias tool (RoB 2) was prespecified for randomized studies; however, it was not applied because no RCTs were included (Table 2).

In addition to conventional quality criteria, the appraisal process considered aspects specific to AI-driven research. These included transparency in AI model development, reporting of training and validation procedures, and clarity regarding model performance metrics (Liu et al. 2020; Hwang et al. 2020). Methodological rigor, such as the use of appropriate control groups and robust statistical analyses, as well as the clinical applicability of reported outcomes, was also evaluated (Zhang and Li 2018; Kim et al. 2017).

NOS ratings indicated predominantly moderate methodological quality. Specifically, 6 studies were rated as high quality (NOS total ≥ 7), 12 as moderate quality (NOS total 5–6), and 3 as low quality (NOS total ≤ 4). Across domains, selection and outcome assessment were commonly adequate, whereas comparability was frequently limited by insufficient control for confounding. Detailed study-level NOS domain scores are provided in Table 3, and the corresponding traffic-light summary and domain-level distribution are presented in Table 4 and 5.

## Discussion

The integration of Artificial Intelligence (AI) into prosthodontic-driven planning for dental implant positioning represents a transformative advancement in contemporary dental practice. Findings from this systematic review affirm AI’s profound capacity to augment diagnostic precision, refine implant placement accuracy, and facilitate highly individualized treatment strategies. Nonetheless, the discourse must be situated within a broader framework that critically addresses technical limitations, ethical considerations, and strategic pathways for clinical translation.

### Enhanced diagnostic capabilities

AI-based image analysis systems may support diagnostic consistency and could help standardize interpretations, thereby potentially reducing inter-operator variability and informing clinical decision-making. In prosthodontic implantology, AI has been increasingly explored as a tool to supplement clinician-dependent image interpretation and to address known limitations related to subjectivity and inter-observer variability in modalities such as cone-beam computed tomography (CBCT) and panoramic radiography (Lee et al. 2019; Yang et al. 2020).

Across the included studies, AI-driven platforms, most commonly those leveraging convolutional neural networks (CNNs), have shown promising performance for radiographic image analysis tasks, including the identification or segmentation of osseous structures, bone density patterns, and key anatomical landmarks such as the inferior alveolar nerve and maxillary sinus (Lee et al. 2019; Yang et al. 2020; Johnson and Blake 2018). These capabilities may improve anatomical mapping and could contribute to more consistent assessments of implant site suitability and risk stratification; however, the extent to which these outputs translate into measurable clinical benefit remains uncertain, given that much of the current evidence derives from observational designs, pilot datasets, and heterogeneous validation approaches.

Studies by Lee et al. and Yang et al. suggest that AI-assisted CBCT interpretation can be associated with fewer interpretation discrepancies and closer agreement with reference standards and/or reported surgical findings in their respective settings (Lee et al. 2019; Yang et al. 2020). Nevertheless, differences in case selection, outcome definitions, and external validation limit generalizability, and causal inferences regarding improved surgical outcomes should be avoided. Overall, integration of AI tools within planning workflows may facilitate greater standardization and reproducibility in image interpretation and pre-operative assessment, but higher-quality prospective and externally validated studies are required to establish clinical effectiveness and impact on patient outcomes (Zhang and Li 2018; Yasui et al. 2021; Garcia et al. 2020; Simmons et al. 2017).

### Reduction in surgical complications

The integration of Artificial Intelligence (AI) into implant surgical workflows has been proposed as a means to support procedural safety by enabling more anatomically detailed and patient-specific preoperative planning. Implant placement entails inherent risks, including neurovascular injury, maxillary sinus perforation, and compromised primary stability, which can be influenced by limitations in preoperative assessment, case complexity, and operator- and workflow-dependent factors (Simmons et al. 2017; Zhou et al. 2020). Within this context, AI-based platforms may assist by applying predictive or decision-support analytics to multidimensional datasets (e.g., bone morphology, occlusal parameters, and soft-tissue features), which could help structure risk stratification and inform surgical planning decisions (Kim et al. 2017; Liu et al. 2020).

Several included studies describe functionalities such as virtual simulation and iterative modification of proposed implant positions or protocols prior to intervention. These capabilities may allow clinicians to identify potential biomechanical or anatomical constraints earlier in the planning process; however, the available evidence is primarily observational and/or pilot in nature, with heterogeneous outcome definitions and limited external validation, which constrains inferences regarding clinical effectiveness.

Overall, current data suggest that AI-assisted planning can be associated with improvements in technical planning or execution metrics (e.g., reduced deviations between planned and placed implants in some settings) and may reduce the need for certain corrective measures (Yasui et al. 2021; Simmons et al. 2017; Zhou et al. 2020). Nevertheless, it remains uncertain whether these findings translate into consistent reductions in iatrogenic complications or improved long-term implant success across broader patient populations. Prospective, adequately powered comparative studies with standardized complication reporting are needed to establish the magnitude and generalizability of any clinical benefit.

### Personalized treatment planning

The increasing use of artificial intelligence (AI) in prosthodontic implantology has been associated with a shift toward more data-informed and individualized planning workflows. Conventional implant planning can be limited in its ability to fully accommodate patient-specific anatomical and biomechanical variability, which may influence prosthetic function and long-term maintenance. In this context, AI-powered systems, using convolutional neural networks, machine learning approaches, and predictive modeling, have been investigated for their capacity to integrate multimodal diagnostic inputs (e.g., CBCT, intraoral scans, and digital occlusal records) and to generate or compare implant placement scenarios in a structured and reproducible manner (Lee et al. 2019; Zhang and Li 2018; Singh and Patel 2019).

Across the included studies, AI tools commonly incorporate patient-level parameters such as bone morphology, occlusal force proxies, and selected systemic factors to produce individualized planning outputs. These outputs may help clinicians consider complex variables, including cortical bone thickness, occlusal considerations, esthetic requirements, and systemic risk factors (e.g., diabetes, smoking), when assessing feasibility and planning strategy (Kim et al. 2017; Liu et al. 2020; Garcia et al. 2020). However, given that much of the available evidence remains observational or pilot in design, claims regarding improved prosthetic longevity or function should be interpreted cautiously, and the degree to which AI-driven planning yields measurable improvements in patient outcomes has not been established consistently.

AI has also been described as a potential adjunct for intraoperative guidance and postoperative monitoring through analytics and feedback mechanisms, but current studies vary substantially in implementation, validation methodology, and endpoint reporting (Yasui et al. 2021; Simmons et al. 2017; Thomas et al. 2019). Similarly, early work on combining AI with robotic-assisted surgery and CAD/CAM prosthodontics suggests potential workflow advantages and technical precision gains in controlled settings, yet broader clinical effectiveness and generalizability remain uncertain (Hwang et al. 2020; Singh and Patel 2019).

While challenges related to dataset representativeness, algorithmic bias, ethical data governance, and clinician adoption remain important (Roberts and Adams 2016; Chen et al. 2019; Blake and Thompson 2018), the reviewed literature indicates that AI may support more personalized planning processes. Future prospective, externally validated studies with standardized clinical endpoints are required to clarify the magnitude of benefit and to determine which patient groups and clinical contexts are most likely to benefit.

### Time and cost efficiency

In addition to potential clinical applications, AI has been increasingly examined for its possible contribution to operational efficiency in prosthodontic implant workflows. Conventional pathways can be time-intensive and resource-demanding, often requiring multiple diagnostic appointments, iterative planning steps, and occasional intraoperative modifications, depending on case complexity and workflow design (Reddy et al. 2019; Blake and Thompson 2018).

AI-driven approaches, particularly automated image analysis, planning support algorithms, and navigation/decision-support functionalities, may help streamline selected steps of this process. For example, Lee et al. reported shorter CBCT analysis/interpretation times using an AI-driven workflow in their study setting (Lee et al. 2019). Similarly, Li et al. described real-time AI assistance during implant surgery as being associated with fewer errors and potential workflow efficiencies, including reduced reliance on manual intraoperative corrections (Yasui et al. 2021). Importantly, these findings derive from individual studies, and no pooled synthesis of time- or cost-related outcomes was feasible due to heterogeneity in AI systems, clinical workflows, operator experience, and endpoint definitions, along with generally small sample sizes and limited external validation. Consequently, any reported efficiency gains should be interpreted as preliminary and not generalizable across clinical environments.

More broadly, the literature suggests that AI may support workflow integration by reducing repetitive planning tasks, facilitating preoperative preparation, and potentially decreasing some postoperative adjustment requirements (Patel et al. 2020; Reddy et al. 2019; Feng et al. 2021). Nevertheless, evidence for downstream economic impact remains limited, and the net effect on costs depends on implementation factors such as acquisition/licensing expenses, training, maintenance, and the extent to which time savings are realized at scale. Prospective comparative studies and health-economic evaluations are needed to clarify whether AI adoption yields consistent reductions in chairside time, resource utilization, or overall treatment costs across different practice settings.

### AI-driven surgical guide design and real-time assistance

The integration of Artificial Intelligence (AI) into preoperative planning and intraoperative support has been increasingly explored in prosthodontic-driven implantology as a means to support procedural precision and workflow predictability. AI-assisted surgical guide design can translate digital treatment plans into clinically deployable guides intended to reproduce planned implant angulation, depth, and spatial alignment. Across the included literature, these approaches are reported to be associated with reduced discrepancies between planned and placed implant positions in certain settings; however, the magnitude of effect varies by guide system, manufacturing workflow, operator experience, and the methods used to define and measure deviation (Patel et al. 2020; Thomas et al. 2019).

Real-time navigation or decision-support systems have also been described as potential adjuncts that analyze intraoperative inputs (e.g., positional tracking data and preoperative imaging registration; in some systems, indirect proxies for anatomical constraints) to provide guidance during execution, particularly in anatomically challenging scenarios such as ridge atrophy or limited restorative space (Yasui et al. 2021; Simmons et al. 2017). Such systems may enable intraoperative adjustments in response to unexpected findings, potentially reducing reliance on static preoperative assumptions. Nonetheless, evidence supporting reductions in complications (e.g., neurovascular injury) remains limited in the current body of predominantly observational and pilot studies, and causal claims should be avoided (Zhang and Li 2018; Simmons et al. 2017; Thomas et al. 2019).

By incorporating patient-specific anatomical information and feedback mechanisms, AI-enabled guidance tools may help reduce operator-dependent variability and could support greater protocol standardization across different levels of experience (Yasui et al. 2021; Simmons et al. 2017; Reddy et al. 2019). At present, however, assertions regarding “higher success rates” or definitive improvements in prosthetic outcomes are not yet substantiated consistently by robust comparative clinical studies. Future research should prioritize prospective designs, standardized reporting of deviation and complication outcomes, and external validation to clarify clinical effectiveness and generalizability.

Despite encouraging early findings, many AI-driven studies remain constrained by risks of algorithmic bias, small or selectively curated datasets, limited external validation, and inconsistent benchmarking. These factors increase the possibility of overfitting and reduce transferability across patient populations, imaging devices, and clinical workflows.

### Clinician over-reliance and the need for human oversight

One of the primary concerns surrounding AI-driven prosthodontic planning is the potential for clinician over-reliance on AI recommendations, which could diminish the role of professional expertise in decision-making. AI models rely on pattern recognition, historical data, and predictive analytics to generate treatment recommendations, but they may lack the nuanced clinical judgment required for complex cases (Roberts and Adams 2016; Reddy et al. 2019).

Roberts and Adams emphasized that AI should be viewed as a decision-support tool rather than a replacement for human expertise, ensuring that prosthodontists remain central in treatment planning and execution (Roberts and Adams 2016). AI-driven models should function as collaborative assistants, augmenting the clinician’s ability to interpret diagnostic data, refine treatment protocols, and improve implant placement precision while still requiring manual oversight and expert intervention in unique or high-risk cases (Reddy et al. 2019; Thomas et al. 2019).

### Ethical and legal considerations: data privacy and algorithm transparency

Beyond the included primary studies, the wider literature highlights ethical and regulatory considerations relevant to AI-enabled implant planning, particularly around data protection, accountability, and explainability. AI systems often require access to large volumes of patient data; accordingly, robust governance frameworks, secure data handling, and transparent audit trails are essential. Within this broader context, blockchain-based approaches have been proposed as one possible mechanism to support record integrity and traceability, but these proposals were not evaluated within the outcomes of the included primary studies and should be interpreted as contextual considerations rather than evidence of clinical effectiveness.

Algorithm transparency (including interpretability of model outputs), clinician oversight, and clear responsibility pathways remain central to ethical implementation. Broader literature commonly frames these issues as prerequisites for trust, informed consent, and regulatory compliance; however, direct comparative evidence on how such governance measures affect implant-planning outcomes was not available within the included primary-study set.

### Integration of AI with augmented reality (AR) and virtual simulations

The integration of AI with augmented reality (AR) and virtual simulation environments has been proposed as a way to enhance visualization and user interaction within digital surgical planning. Although conceptually appealing, AR-enabled workflows were not directly assessed as outcome-bearing interventions in the included primary studies; therefore, discussion of AR here is presented as horizon scanning rather than as a finding of the systematic review.

In principle, AR interfaces could support communication of planned implant positioning by superimposing virtual information onto patient-specific anatomy, potentially aiding spatial understanding and intraoperative orientation. At present, however, the available reports are heterogeneous and often focus on feasibility, educational applications, or technical demonstrations, limiting any inference about routine clinical benefit in prosthodontic-driven implant planning.

Where AI-enhanced simulation tools have been described in dental training contexts, the reported outcomes typically relate to learner experience or task performance in controlled settings rather than patient outcomes. Extrapolation of such results to clinical implant planning should therefore be made cautiously, and prospective clinical evaluations would be required to establish effectiveness, safety, and implementation requirements.

If developed and validated appropriately, AR-linked planning systems might enable real-time visualization of relevant anatomical structures and planned implant trajectories. Nevertheless, empirical evidence demonstrating improvements in complication rates, long-term implant success, or prosthetic outcomes remains limited, and high-quality comparative studies are needed before these approaches can be considered evidence-based adjuncts in routine care.

### Future AI applications: predictive analytics and prosthodontic treatment optimization

Looking forward, broader literature frequently positions AI development toward predictive analytics, decision support, and multi-parameter optimization across prosthodontic-driven implant workflows. These directions are plausible given advances in data integration and model performance; however, many proposed applications remain at an early stage, and their clinical value will depend on external validation, standardized endpoint reporting, and demonstrated benefit in prospective comparative studies.

Within these emerging directions, AR interfaces and blockchain-based data-governance frameworks have been discussed as potential enablers for enhanced visualization and strengthened data traceability. These topics were not evaluated as outcomes within the included primary studies and should be interpreted as implications for future research and implementation planning. Any integration with CAD/CAM systems and occlusal analysis platforms likewise requires rigorous validation, transparent reporting, and appropriate regulatory oversight to ensure reliability, accountability, and equitable access.

### Limitations

Across the included studies, reporting of AI-specific methodological features was frequently incomplete and heterogeneous, limiting assessment of model validity and transferability. Several studies provided limited detail regarding training dataset provenance and size, inclusion/exclusion criteria, class balance, preprocessing and augmentation steps, and procedures to prevent data leakage. External validation was inconsistently performed; many models were evaluated primarily using internal splits (e.g., training/validation/test partitions within the same dataset) rather than independent datasets from different institutions, scanners, or patient populations. In addition, generalizability was rarely examined through multi-center evaluation, device/domain shift testing, or subgroup analyses relevant to implant planning (e.g., variations in bone quality, artifact burden, edentulism patterns, or restorative space constraints). Reporting of performance metrics also varied, with inconsistent use of confidence intervals, calibration measures, and clinically interpretable endpoints. Collectively, these limitations constrain comparability across studies and reduce certainty regarding real-world clinical performance, underscoring the need for standardized AI reporting and prospective validation frameworks in implant planning applications.

## Conclusion

Artificial intelligence (AI) represents a rapidly developing component of prosthodontic-driven implantology, with potential implications for planning precision, workflow efficiency, and individualized treatment strategies. When applied with appropriate clinician oversight, empirical validation, and ethical governance, AI may contribute to the evolution of contemporary prosthodontic practice. This systematic review highlights current trends suggesting that AI-assisted tools can support diagnostic standardization, planning consistency, and data-informed clinical decision-making, while acknowledging that evidence regarding direct clinical outcomes remains heterogeneous.

Overall, the available literature indicates that AI has the potential to support prosthodontic-driven dental implantology by providing clinicians with advanced analytical tools that may enhance implant planning accuracy, assist in risk assessment, and facilitate individualized care. Importantly, AI should be integrated into clinical workflows as a collaborative technology rather than a substitute for professional judgment. When used in this capacity, AI may contribute to improved predictability and efficiency of implant treatment, provided its limitations are clearly recognized and addressed.

The integration of AI into prosthodontic-driven implantology reflects a multidimensional shift across diagnostic, planning, and evaluative phases of care. As an adaptive and supportive technology, AI may assist clinicians in delivering more personalized, evidence-based treatment by improving workflow organization and supporting clinical decision-making. Ensuring that AI functions as a complementary partner to human expertise will be essential as the field progresses toward a future characterized by intelligent, patient-centered, and precision-oriented prosthodontic care.

## Data Availability

The data supporting the outcomes of this study are available from the corresponding author upon reasonable request.

## Appendix A: Tables

**Table 1:** Structured comparison of artificial intelligence (AI) applications across prosthodontic treatment phases, highlighting key functionalities, clinical advantages, and supporting evidence from included primary studies

Treatment Phase	AI Application	Key Functionalities	Clinical Benefits	Notable References	Study Design/Level of Evidence*
**1. Diagnostic Phase**	AI-Assisted Radiographic Interpretation	Analysis of CBCT, panoramic X-rays, and intraoral scans using CNNs and deep learning algorithms	Increased diagnostic precision, reduced inter-clinician variability, early detection of pathologies	(Lee et al. 2019; Yang et al. 2020; Johnson and Blake 2018)	Level III–IV (predominantly diagnostic accuracy/observational; some methodological/technical)
	Anatomical Landmark Detection	Automatic identification of inferior alveolar nerve, sinus floor, and cortical boundaries	Potential reduction in anatomical violation risk; improved implant site assessment	(Lee et al. 2019; Patel et al. 2020; Johnson and Blake 2018; Simmons et al. 2017)	Level III–IV (diagnostic accuracy/observational imaging studies)
**2. Treatment Planning**	AI-Based Predictive Modeling	Evaluation of bone quality, occlusal load, systemic risk factors, and soft tissue characteristics	Individualized risk stratification, optimized implant trajectory, potential prognosis support	(Kim et al. 2017; Liu et al. 2020)	Level III–IV (observational/model development with clinical datasets)
	Virtual Implant Simulation	Real-time modeling of implant angulation, depth, spacing, and prosthetic alignment	Potential reduction in planning errors; improved prosthetic fit predictability	(Yasui et al. 2021)	Level IV (pilot/feasibility clinical study)
	CAD/CAM Integration	AI-driven prosthetic design based on esthetic, biomechanical, and occlusal parameters	Improved esthetic outcomes; functionally driven restorations	(Blake and Thompson 2018)	Level IV (practice-oriented clinical/technical report)
**3. Surgical Phase**	AI-Guided Surgical Guide Design	Automated translation of digital plans into surgical templates	High-precision placement, fewer intraoperative adjustments	(Patel et al. 2020)	Level III (clinical accuracy/outcomes study)
	AI-Enhanced Navigation & Robotics	Real-time intraoperative feedback using AI and robotic assistance	Enhanced surgical accuracy; reduced deviations; potentially safer execution in complex cases	(Yasui et al. 2021; Simmons et al. 2017)	Level IV (pilot/observational clinical studies)
**4. Follow-Up Phase**	AI-Based Prognostic Monitoring	Monitoring bone remodeling, prosthetic wear, and occlusal force changes over time	Early detection of complications; proactive maintenance strategies (potential)	(Feng et al. 2021; Liu et al. 2020)	Level III–IV (observational prognostic modeling/clinical datasets)

**Table 2:** Prespecified Cochrane risk of bias domains for randomized trials (RoB 2)

RoB tool	Domain-level judgments
Cochrane RoB 2 (RCTs)	Not applicable in this review because no randomized controlled trials were included. Prespecified domains: bias arising from the randomization process; deviations from intended interventions; missing outcome data; measurement of the outcome; selection of the reported result. Overall judgments would be classified as low risk, some concerns, or high risk.

**Table 3:** Risk-of-bias assessment of included studies using the Newcastle–Ottawa scale (NOS)

Study ID	Selection (0–4)	Comparability (0–2)	Outcome (0–3)	NOS Total (0–9)	Overall judgment
Study 1	4	2	2	8	High
Study 2	4	1	3	8	High
Study 3	3	2	3	8	High
Study 4	4	1	2	7	High
Study 5	3	2	2	7	High
Study 6	4	0	3	7	High
Study 7	3	1	2	6	Moderate
Study 8	3	0	3	6	Moderate
Study 9	3	1	1	5	Moderate
Study 10	2	1	2	5	Moderate
Study 11	3	0	2	5	Moderate
Study 12	2	2	1	5	Moderate
Study 13	3	1	2	6	Moderate
Study 14	3	0	3	6	Moderate
Study 15	3	1	1	5	Moderate
Study 16	2	1	2	5	Moderate
Study 17	3	0	2	5	Moderate
Study 18	2	2	1	5	Moderate
Study 19	2	1	1	4	Low
Study 20	2	0	2	4	Low
Study 21	1	1	1	3	Low

**Table 4:** Traffic-light plot and domain-level distribution of NOS risk-of-bias judgments across included studies

Study ID	Selection	Comparability	Outcome
Study 1	Low	Low	Some concerns
Study 2	Low	Some concerns	Low
Study 3	Some concerns	Low	Low
Study 4	Low	Some concerns	Some concerns
Study 5	Some concerns	Low	Some concerns
Study 6	Low	High	Low
Study 7	Some concerns	Some concerns	Some concerns
Study 8	Some concerns	High	Low
Study 9	Some concerns	Some concerns	High
Study 10	High	Some concerns	Some concerns
Study 11	Some concerns	High	Some concerns
Study 12	High	Low	High
Study 13	Some concerns	Some concerns	Some concerns
Study 14	Some concerns	High	Low
Study 15	Some concerns	Some concerns	High
Study 16	High	Some concerns	Some concerns
Study 17	Some concerns	High	Some concerns
Study 18	High	Low	High
Study 19	High	Some concerns	High
Study 20	High	High	Some concerns
Study 21	High	Some concerns	High

**Table 4:** (continued). Domain-level distribution of judgments (n=21 studies)

Domain	Low (n)	Some concerns (n)	High (n)
Selection	4	10	7
Comparability	5	10	6
Outcome	5	10	6

## Abbreviations

AI: Artificial Intelligence
AR: Augmented Reality
